# Blue light-dependent changes in loosely bound calcium in Arabidopsis mesophyll cells: an X-ray microanalysis study

**DOI:** 10.1093/jxb/erw089

**Published:** 2016-03-08

**Authors:** Justyna Łabuz, Sławomir Samardakiewicz, Paweł Hermanowicz, Elżbieta Wyroba, Maria Pilarska, Halina Gabryś

**Affiliations:** ^1^Department of Plant Biotechnology, Faculty of Biochemistry, Biophysics and Biotechnology, Jagiellonian University, Krakow, Poland; ^2^Malopolska Centre of Biotechnology, Jagiellonian University, Krakow, Poland.; ^3^Laboratory of Electron and Confocal Microscopy, Faculty of Biology, Adam Mickiewicz University, Poznań, Poland.; ^4^Laboratory of Electron Microscopy, Nencki Institute of Experimental Biology, Polish Academy of Sciences, Warsaw, Poland

**Keywords:** *Arabidopsis*, *thaliana*, blue light, calcium signaling, chloroplast movements, mesophyll cells, phototropin2.

## Abstract

Calcium is involved in the signal transduction pathway from phototropins, the blue light photoreceptor kinases which mediate chloroplast movements. The chloroplast accumulation response in low light is controlled by both phot1 and phot2, while only phot2 is involved in avoidance movement induced by strong light. Phototropins elevate cytosolic Ca^2+^ after activation by blue light. In higher plants, both types of chloroplast responses depend on Ca^2+^, and internal calcium stores seem to be crucial for these processes. Yet, the calcium signatures generated after the perception of blue light by phototropins are not well understood. To characterize the localization of calcium in Arabidopsis mesophyll cells, loosely bound (exchangeable) Ca^2+^ was precipitated with potassium pyroantimonate and analyzed by transmission electron microscopy followed by energy-dispersive X-ray microanalysis. In dark-adapted wild-type Arabidopsis leaves, calcium precipitates were observed at the cell wall, where they formed spherical structures. After strong blue light irradiation, calcium at the apoplast prevailed, and bigger, multilayer precipitates were found. Spherical calcium precipitates were also detected at the tonoplast. After red light treatment as a control, the precipitates at the cell wall were smaller and less numerous. In the *phot2* and *phot1phot2* mutants, calcium patterns were different from those of wild-type plants. In both mutants, no elevation of calcium after blue light treatment was observed at the cell periphery (including the cell wall and a fragment of cytoplasm). This result confirms the involvement of phototropin2 in the regulation of Ca^2+^ homeostasis in mesophyll cells.

## Highlight

Localization of loosely bound calcium in Arabidopsis mesophyll changes under strong blue light in the wild type, but not in *phot2* and *phot1phot2* mutants. This indicates that phot2 is involved in calcium homeostasis.

## Introduction

Calcium ions are considered to be an extremely versatile secondary messenger, a key element of many responses to biotic and abiotic factors (for a review, see Dodd *et al.*, 2010). In plants, specific ‘calcium signatures’ are generated, mainly in the cytosol, but also in the nucleus, mitochondria, and chloroplasts (Stael *et al.*, 2012). The precise control of Ca^2+^ translocations between organelles and the apoplast generates spatially and temporally distinct cytosolic calcium patterns and thus produces stimulus-specific responses (McAinsh and Pittman, 2009).

In the case of blue light signaling, calcium elevation follows the activation of phototropins. Phototropins are UVA and blue light photoreceptor kinases which mediate plant movements and rapid growth responses. The two phototropins of *Arabidopsis thaliana*, phot1 and phot2, are characterized by different light sensitivities, though they share highly redundant functions. They both control phototropism, leaf expansion, stomatal opening, and the chloroplast accumulation response. Only phot1 mediates the inhibition of the hypocotyl growth reaction, and only phot2 mediates chloroplast avoidance and dark positioning (for a review, see Christie, 2007). Although both Arabidopsis phototropins are responsible for Ca^2+^ mobilization after blue light treatment, differences in the localization of cellular calcium signals depending on light intensity, plant organ, and the phototropin itself have been reported (Harada and Shimazaki, 2007).

In 10- to 16-day-old Arabidopsis seedlings, a pulse of very strong blue light (10s, 600 μmol m^−2^ s^−1^) resulted in a transient increase in cytosolic calcium concentration, [Ca^2+^]_cyt_, which diminished after treatment with lanthanum (La^3+^) ions (calcium channel inhibitors). In *phot1* mutant seedlings, [Ca^2+^]_cyt_ was increased in darkness, but the elevation after blue light treatment was half that of the wild type. This indicated the role of phot1 in mobilizing Ca^2+^ from intercellular spaces through cell membrane-localized channels (Baum *et al.*, 1999). In 3-day-old etiolated Arabidopsis wild-type seedlings, an increase in calcium concentration was observed after a pulse of blue light (10s, 100 μmol m^−2^ s^−1^). This response was inhibited by BAPTA (a Ca^2+^ chelator), similarly to the inhibition of hypocotyl growth, controlled by phot1 (Folta *et al.*, 2003). In 4-day-old etiolated Arabidopsis wild-type seedlings, the illumination of hypocotyls and cotyledons with continuous blue light (25 μmol m^−2^ s^−1^) for 10min caused a transient increase in [Ca^2+^]_cyt_. In wild-type and *phot2* mutant plants, the biggest influx of calcium was detected in the third minute after turning on the light. In the *phot1* mutant, a residual effect was present and, since it was not observed at all in the double *phot1phot2* mutant, calcium mobilization from the apoplast was attributed to phot1 (Babourina *et al.*, 2002). A potential-gated cation channel called PACC, a phototropin-activated calcium-permeable channel, was identified in mesophyll protoplasts based on the observation of a Ca^2+^ flux after continuous illumination with blue light of 275 μmol m^−2^ s^−1^. This process became saturated after 11–16min of irradiation and was inhibited by La^3+^ and protein kinase inhibitors. In the *phot1* mutant, the activity of this channel was greatly reduced and it was not detected in the double *phot1phot2* mutant (Stoelzle *et al.*, 2003).

In 3-week-old Arabidopsis rosette leaves, changes in [Ca^2+^]_cyt_ were observed after 10s blue light pulses. In wild-type plants, an increase in calcium concentration occurred at light intensities of 0.1–250 μmol m^−2^ s^−1^, phot1 being active in the range of 0.1–50 μmol m^−2^ s^−1^ and phot2 in the range of 1–250 μmol m^−2^ s^−1^. Calcium channel inhibitors, Co^2+^, La^3+^, and nifedipine, and calcium chelators, EGTA and BAPTA, caused a significant reduction in the blue light response in wild-type plants and phototropin mutants, indicating a mechanism of Ca^2+^ influx into the cytoplasm through membrane channels regulated by both photoreceptors. On the other hand, phospholipase C inhibitors neomycin and U-73122 inhibited the blue light increase in [Ca^2+^]_cyt_ in wild-type and *phot1* mutant plants, but not in the *phot2* mutant. Thus phot2 was suggested to be responsible for the phospholipase C-dependent release of Ca^2+^ from internal stores, such as the vacuole or endoplasmic reticulum (ER) (Harada *et al.*, 2003).

The role of calcium in the control of chloroplast movements was proposed long before the discovery of phototropins (for a review, see Banaś *et al.*, 2012). The rotation of the flat, ribbon-like chloroplast of the filamentous alga *Mougeotia* was shown to depend on the presence of specialized membrane vesicles which contain calcium ions (Wagner and Klein, 1981). However, other works using calcium inhibitors suggested that the disruptions in chloroplast rotation resulted from toxic effects or disturbances in processes other than the photosensory transduction chain (Schönbohm *et al.*, 1990). In *Vallisneria gigantea*, the movement of chloroplasts under red light correlated with cytoplasmic streaming within the cell and was precisely regulated by Ca^2+^ concentration (Takagi and Nagai, 1983). In *Physcomitrella patens* (Sato *et al.*, 2003) and *Adiantum capillus-veneris* (Sato *et al.*, 2001), external calcium was involved in triggering chloroplast movement induced by mechanical stimulation. Inhibitors of calcium channels (La^3+^ and Ga^3+^) did not affect the photo-relocation of chloroplasts in these species.

Two types of blue light-controlled chloroplast movements have been characterized in higher plants. One is the accumulation response observed in Arabidopsis under low blue light (0.08–4 μmol m^−2^ s^−1^). Chloroplasts move to cell walls lying perpendicular to the direction of incident light. The other is the avoidance response. Under strong blue light (>20 μmol m^−2^ s^−1^), chloroplasts gather under walls parallel to the direction of incident light (Trojan and Gabryś, 1996; Sakai *et al.*, 2001).

The involvement of calcium in the control of blue light-induced chloroplast relocations was put forward on the basis of inhibitor treatments in the aquatic angiosperm *Lemna trisulca* (Tlałka and Gabrys, 1993; Tlałka and Fricker, 1999). A prolonged incubation (12h) with EGTA was needed for a partial inhibition of chloroplast avoidance. However, in tissues initially pre-treated with the A23187 ionophore, only 1h long EGTA incubation was sufficient to disturb chloroplast movements. Similarly, A23187 pre-treatment followed by 1h incubation with lanthanum enhanced its inhibitory effect on chloroplast relocations. These observations indicated the key role of internal calcium stores in the control of chloroplast movements (Tlałka and Gabrys, 1993). This hypothesis was supported by strong inhibitory effects observed after 2min treatments with caffeine (which causes a release of calcium from intracellular stores) and thapsigargin (a selective inhibitor of the ER Ca^2+^-ATPase). The incubation of *Lemna* fronds with calcium channel inhibitors nifedipine (for 3h) and verapamil (for 1h) also partially disturbed chloroplast movements (Tlałka and Fricker, 1999).

In *Nicotiana tabacum*, the exogenous application of Ca^2+^ did not affect movements. However, the disturbance in calcium homeostasis by incubating the tissue with the A23187 ionophore proved detrimental to chloroplast relocations. In tobacco, in contrast to duckweed, short-term treatment with EGTA or TFP (trifluoperazine; an inhibitor of calmodulin) was sufficient to inhibit both chloroplast responses strongly. This effect was at least partially caused by disorders in the actin cytoskeleton, which is indispensable for movements. The subsequent addition of Ca^2+^ partially restored the ability of chloroplasts to move (Anielska-Mazur *et al.*, 2009). A similar movement reactivation after EGTA treatment was observed in *Adiantum* (Kadota and Wada, 1992). Inhibition by TFP implicated a calmodulin-dependent signal transduction pathway, consistent with previous findings on chloroplast rotation control in *Mougeotia* (Wagner *et al.*, 1984).

Indirect evidence for the role of calcium in the control of chloroplast relocations in Arabidopsis came from a study showing the involvement of phosphoinositides in the blue light signaling pathway. The phospholipase C pathway was suggested to take part in phot2 signaling, while the phosphatidylinositol kinases PI3K and PI4K were suggested to control the accumulation response mediated by both phototropins. U73-122 (the phospholipase C inhibitor) as well as wortmannin and LY294002 (two inhibitors of the PI3K pathway) suppressed the transient calcium elevation induced by blue light in Arabidopsis leaves (Aggarwal *et al.*, 2013*a*, *b*). In line with that, the inhibitory effect of wortmannin on chloroplast movements in *Nicotiana* could be over-ridden by the application of external Ca^2+^ (Anielska-Mazur *et al.*, 2009).

Even though substantial evidence has been collected, the role of calcium in phototropin-controlled physiological responses needs further investigation. Ca^2+^ fluxes have been analyzed by aequorin luminescence (Baum *et al.*, 1999; Harada *et al.*, 2003), ion-selective microelectrode (Babourina *et al.*, 2002), and patch-clamp methods (Stoelzle *et al.*, 2003). These show changes in calcium concentration mainly as a function of time. Although indirect evidence has been obtained using inhibitors, differences in the spatial distribution of Ca^2+^ within the cell and the relevant calcium storage compartments remain to be specified. Studies on calcium in Arabidopsis leaves (Harada *et al.*, 2003) and mesophyll protoplasts (Stoelzle *et al.*, 2003) determine light ranges in which phot1 and phot2 affect Ca^2+^ concentrations; however, they do not answer the question of how these changes correspond to chloroplast movement signaling. Spatial and temporal aspects of calcium release are important to understand how specific calcium signatures are generated. In this work, Ca^2+^ precipitation with KPA (potassium pyroantimonate) and transmission electron microscopy (TEM) followed by X-ray microanalysis were employed to study changes in calcium distribution. The main aim was to determine the localization of Ca^2+^ after blue light treatment in Arabidopsis mesophyll cells in order to elucidate how calcium participates in directing chloroplast movements. The intensity of blue light and the duration of irradiation were chosen to investigate the phot2-specific chloroplast avoidance response.

## Materials and methods

### Plant material and growth conditions

Seeds of Arabidopsis wild-type Columbia were obtained from Nottingham Arabidopsis Stock Centre (Nottingham, UK). Mutant seeds were the kind gifts of A.R. Cashmore, the Plant Science Institute, Department of Biology, University of Pennsylvania, Philadelphia, USA (*phot2*) and J. Jarillo, Instituto Nacional de Investigación y Tecnología Agraria y Alimentaria, Madrid, Spain (*phot1phot2*). Plants were grown in a growth chamber (Sanyo MLR 350H, Japan) with a 10h/14h light/dark cycle at 23 °C, with 80% relative humidity, and illuminated by ﬂuorescent lamps (FL40SS.W/37, Sanyo, Japan) with a photosynthetic photon ﬂux density of 60–100 μmol m^−2^ s^−1^.

### Tissue processing

Five-week-old plants were dark-adapted for at least 12h. The fifth or sixth rosette leaf was irradiated directly on the plant for 3min with blue light (LXHL-PR09, Ledium Ltd, Hungary) of 100 μmol m^−2^ s^−1^ to induce the phot2-mediated chloroplast avoidance response. Control plants were kept untreated in darkness or were irradiated for 3min with equimolar red light (LXHL-PD09, Ledium Ltd), which does not activate phototropins. Immediately after treatment, leaves were cut into 2mm strips and put directly into the fixative solution. A suitable cutting margin was taken into account, so that the wounded site did not lie adjacent to the cells used for analysis. In order to minimize calcium elution, pyroantimonate precipitation was performed concomitantly with material fixing. Tissue sections were infiltrated with a syringe containing a fixative solution of 2% glutaraldehyde, 2% potassium pyroantimonate in a phosphate buffer (100mM KH_2_PO_4_/K_2_HPO_4_, pH 7.4) at 4 °C (according to Tretyn *et al.*, 1992; Musetti and Favali, 2003) and incubated on ice for 2h. All steps were performed in darkness, using only ‘safe’ green light. After fixation, the material was washed four times in a chilled phosphate buffer (100mM KH_2_PO_4_/K_2_HPO_4_, pH 7.6, 3×10min, 1×15min) and subsequently stained with 1% osmium tetra-oxide in a 100mM phosphate buffer at 4 °C. After dehydration in an acetone series (10, 30, 50, 70, 90, 96, 100%, 2×5min), the material was embedded in epoxy resin of low viscosity (Spurr, 1969). Cross-sections of leaves (100nm thick) were obtained with an EM-U-C6 ultramicrotome (Leica, Austria) and put on copper grids coated with formvar and carbon.

### TEM and X-ray microanalysis

**Fig. 1.**
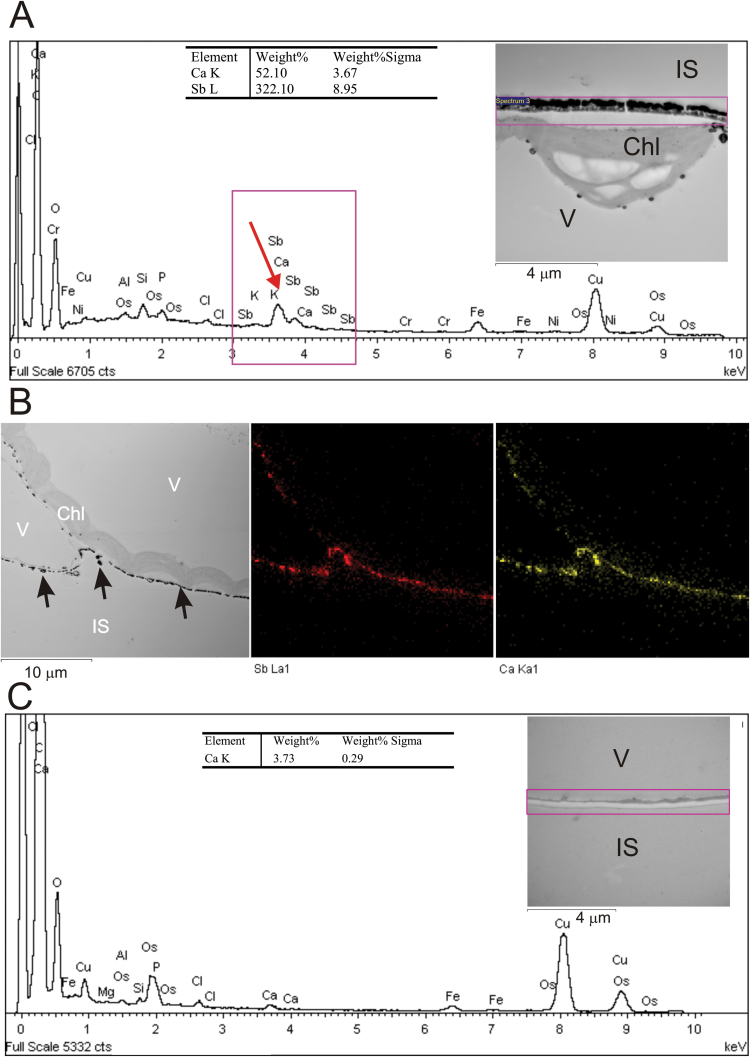
(A) Example X-ray spectra obtained from a selected rectangular region of the periphery of an Arabidopsis wild-type mesophyll cell (including the cell wall and a fragment of cytoplasm), which contains precipitates of calcium and antimony. The relative content (Weight %) of individual elements was calculated on the basis of the peak area (arrow) characteristic of the calcium–Kα line (Ca K) and antimony–Lα line (Sb L). The error value quoted is sigma, which is the statistical error for the calculated Wt%. (B) An example map showing calcium (yellow) and antimony (red) localization within the analyzed cells. (C) Control X-ray spectra obtained from a selected rectangular region of the periphery of a mesophyll cell (including the cell wall and a fragment of cytoplasm) not treated with potassium pyroantimonate. Chl, chloroplast; IS, intercellular space; V, vacuole.

Leaves were harvested from at least two (2–4) different batches of plants. Samples were analyzed under a JEM 1400 transmission electron microscope (JEOL Co., Japan) equipped with a Morada CCD camera (SiS-Olympus, Japan) at an accelerating voltage of 80 keV. The X-ray microanalysis was performed using the energy-dispersive full range X-ray microanalysis system EDS INCA Energy TEM (Oxford Instruments, UK). The detection and semi-quantitative analysis of calcium and antimony were carried out by collecting X-ray spectra from a selected region of interest in the energy range of 1–10keV (Fig. 1). The relative content of calcium and antimony was calculated using standards on the basis of the peak area characteristic of the calcium–K_α_ emission line (3.691 keV) and antimony–L_α_ emission line (3.605 keV), with Oxford INCA TEM 200 software. The identification and localization of calcium precipitates in cells was determined by mapping the distribution of the element in certain parts of the tissue (X-ray mapping). The semi-quantitative analysis was carried out by measuring the spectra from identical squares (10 µm^2^) for 200s, at ×15 000 magnification, from the peripheral areas of mesophyll cells including the cell wall and a fragment of cytoplasm (Fig. 1). The regions inside the vacuole and intercellular space, which rarely contained visible precipitates, were chosen as controls. The data on content of elements were processed using EDS INCA software for the assessment of the percentage weight concentration of elements after correction for interelement effects (Wt%). Spectra were recorded in at least five (5–9) different regions of the leaf mesophyll. A 3D model of precipitates was created with a tomographic holder and Chimera software. The surface area for precipitate cross-sections was measured with the particle analysis procedure available in ImageJ. Particle analysis was performed on images showing leaf mesophyll (magnifications between ×400 and ×1020), which were manually segmented into cell regions (cell wall, vacuole, and tonoplast). The surface area of precipitates adjacent to the outer face of the cell wall was expressed per unit of the cell wall length, while the area of precipitates localized in the vacuole at the tonoplast was expressed per unit of the tonoplast length. The significance of the effects of light conditions and the plant line on the mean calcium content and precipitate area was assessed with ANOVA. For pairwise comparison of means, Tukey’s test was performed after one-way ANOVA, calculated separately for each plant line. Adjusted *P*-values from Tukey’s test are indicated in the figures with asterisks. The tests were performed with R-software.

## Results

The KPA precipitation method does not depict the overall distribution of calcium within cells, but only that fraction which is susceptible to precipitation. Any calcium present in an appropriate concentration, not too tightly bound to cellular organelles and not readily washed out during the procedure, may be analyzed. The analysis of KPA precipitates yields information about the difference in the distribution and content of calcium dependent on a given factor, so the interpretation of results should be done on the basis of controls. In our experimental model, Arabidopsis leaves were exposed to blue light of 100 μmol m^−2^ s^−1^ for 3min to activate phot2 and induce a measurable chloroplast avoidance response. In wild-type plants, this time point corresponds to the moment when chloroplasts achieve maximum velocity after the onset of strong blue light (Łabuz *et al.*, 2015). Control leaves were incubated in the dark or irradiated with equimolar red light which does not affect phototropins. Carefully selected conditions, including the use of a phosphate buffer with a slightly alkaline pH along with glutaraldehyde fixation, were optimized for the formation of pyroantimonate precipitates only with calcium ions, while reducing the specificity of the reaction to monovalent ions and Mg^2+^ (Wick and Hepler, 1982). Spectra (Fig. 1A) and maps (Fig. 1B) of relevant cell areas were collected in order to confirm the co-localization of calcium (marked yellow) with antimony (marked red) in these electron-dense structures. A control spectrum showing the element composition in cells treated without KPA is shown in Fig. 1C.

### The localization of calcium in the mesophyll cells of wild-type Arabidopsis

**Fig. 2.**
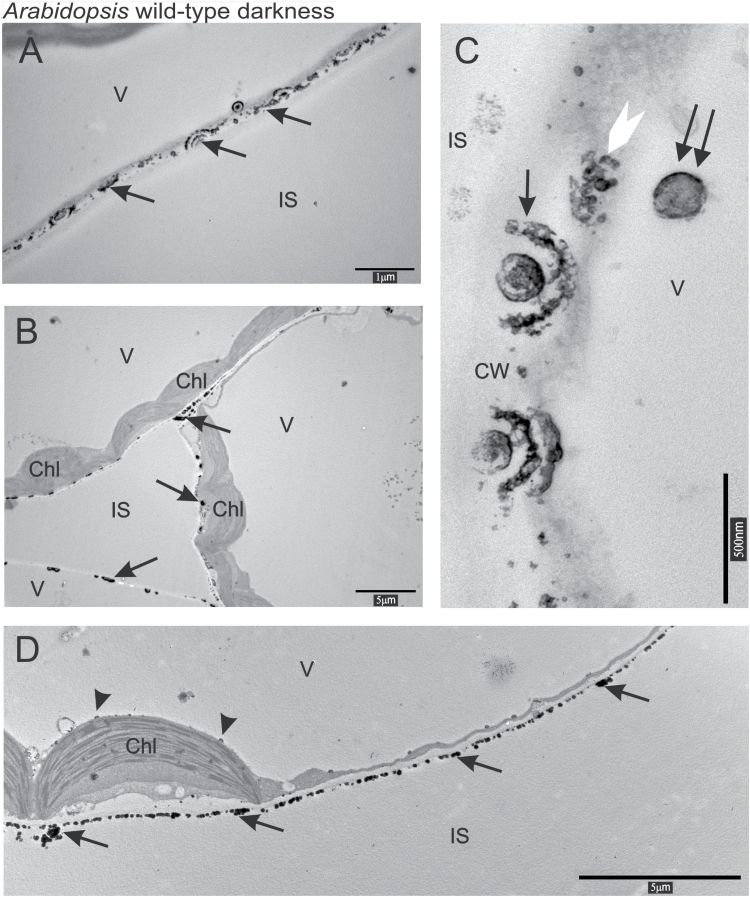
(A–D) The localization of calcium and antimony precipitates in Arabidopsis wild-type mesophyll cells in darkness. Arrows indicate precipitates of calcium and antimony at the cell wall; the white arrowhead precipitates in the cytosol; a double arrow precipitates in the vacuole; and black arrowheads precipitates adjacent to the chloroplast envelope and the tonoplast. CW, cell wall, Chl, chloroplast; IS, intercellular space; V, vacuole.

In the mesophyll cells of dark-adapted Arabidopsis wild-type leaves, calcium precipitates formed circular, semi-circular, or lenticular structures of different sizes (Fig. 2A–D). At the cell wall these spherical structures had several layers concentrically propagating in both directions, from and into the cell (Fig. 2A, C). Precipitates of higher densities were observed along the cell walls (Fig. 2D). Circular precipitates and very small granules were occasionally found in the cytosol, in the vacuole (Fig. 2C), and at the chloroplast envelope outer membrane (Fig. 2D).

**Fig. 3.**
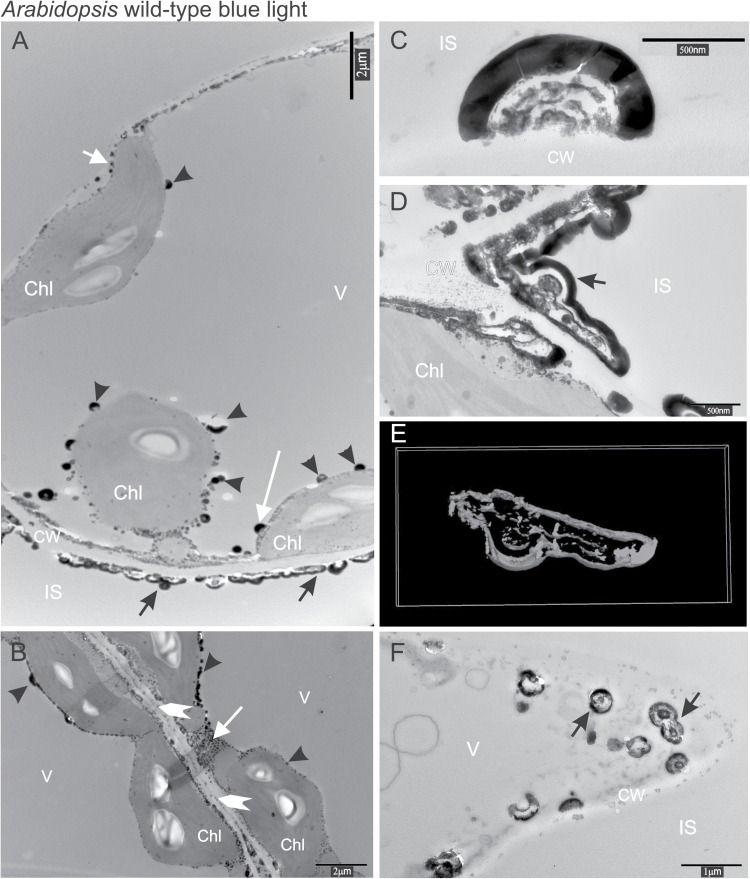
(A, B) The localization of calcium and antimony precipitates in Arabidopsis wild-type mesophyll cells after 3min blue light treatment of 100 μmol m^−2^ s^−1^. Cross-section of the precipitates (C, D) perpendicular and (F) parallel to the cell wall plane. (E) The 3D model of the precipitate created from (D). Arrows indicate precipitates of calcium and antimony at the cell wall; white arrowheads precipitates in the middle lamella; and black arrowheads precipitates adjacent to the chloroplast envelope and the tonoplast. A long white arrow indicates precipitates on the tonoplast directed towards the vacuole interior; a short white arrow precipitates on the outer membrane of the chloroplast envelope pointing towards the cytosol; and a medium sized arrow indicates precipitates in the cytosol. CW, cell wall, Chl, chloroplast; IS, intercellular space; V, vacuole.

In cells irradiated with blue light for 3min, the calcium localization pattern was similar to that in darkness. However, the size and density of structures in the regions of cell walls facing the intercellular spaces were significantly increased (Fig. 3A, D). There were relatively few precipitates in the middle lamella (Fig. 3B). Single (Fig. 3C, F) and clustered (Fig. 3A, D) structures were found along the edges of cell walls. Some of these were surrounded by dark gray or black bands of varying thickness arranged in several concentric layers (Fig. 3C, D). A typical picture of cells after blue light treatment showing the connected precipitates which align along the cell wall is shown in Fig. 3A. Analysis of cell wall cross-sections revealed that calcium precipitates were formed by overlapping hemispheres (Fig. 3F). This observation was confirmed by a 3D model based on tomography images. The 3D structure was limited by the thickness of the slice (100nm), but it demonstrated that cross-sections indeed represent the segments of a sphere (Fig. 3D, E). In areas where precipitates were rarely seen in dark conditions, such as the cytosol (Fig. 3B, white arrow), tonoplast, and the chloroplast envelope (Fig. 3A), they became more abundant after blue light treatment (Fig. 3A, B, D). Semi-circular or lenticular precipitates on the tonoplast were oriented towards the vacuole interior (Fig. 3A, long white arrow) and those observed on the outer membrane of the chloroplast envelope pointed towards the cytosol (Fig. 3A short white arrow).

**Fig. 4.**
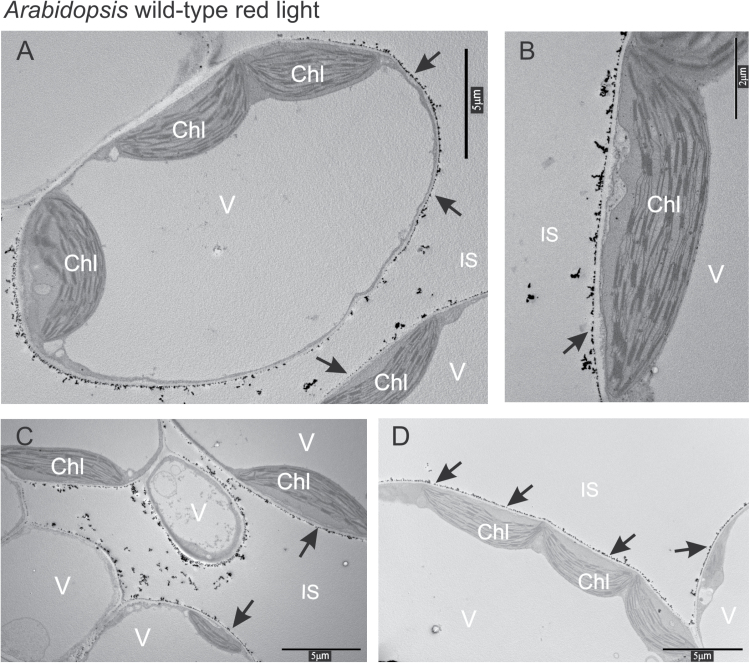
(A–D) The localization of calcium and antimony precipitates in Arabidopsis wild-type mesophyll cells after 3min red light treatment of 100 μmol m^−2^ s^−1^. Arrows indicate precipitates of calcium and antimony at the cell wall. Chl, chloroplast; IS, intercellular space; V, vacuole.

After 3min of red light treatment, the precipitates at the cell wall were much smaller and did not form the characteristic spherical structures (Fig. 4A–D) as compared with darkness and blue light. No precipitates were observed in the cytosol, on the tonoplast, and on the outer membrane of the chloroplast envelope (Fig. 4A, B).

### The localization of calcium in the mesophyll cells of Arabidopsis phototropin mutants

**Fig. 5.**
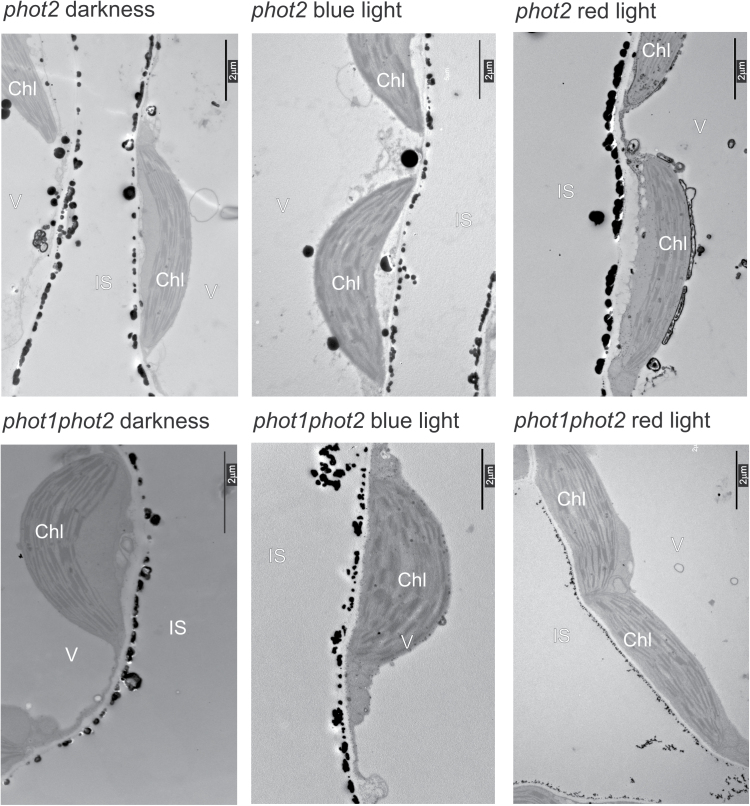
The localization of calcium and antimony precipitates in mesophyll cells of the Arabidopsis phototropin mutants *phot2* and *phot1phot2* in darkness, after 3min of blue or red light treatment of 100 μmol m^−2^ s^−1^. Chl, chloroplast; IS, intercellular space; V, vacuole.

In order to link the differences in Ca^2+^ patterns observed after light treatments with phot2 signaling, the localization of calcium precipitates in the *phot2* and *phot1phot2* mutants was investigated. Only phot2 is responsible for calcium mobilization in mature Arabidopsis leaves under the light conditions used in this study (100 μmol m^−2^ s^−1^), as demonstrated by Harada *et al.* (2003). Figure 5 shows the localization of calcium precipitates in the cells of the phototropin mutants. Generally, a smaller variation in calcium structures at the cell wall was observed in these mutants. Calcium precipitates forming spherical structures were rarely multilayered. In dark conditions, the *phot2* and *phot1phot2* mutants had precipitates with a firm ‘bead’ structure, which usually merged into bands running along the cell walls, as compared with the Arabidopsis wild type (compare Figs 2 and 5 *phot2* darkness, *phot1phot2* darkness). In the *phot2* mutant, structures at the cell wall under blue light and red light were similar to those observed under dark conditions. Precipitates found in *phot2* after red light had a defined spherical structure, in contrast to those observed in wild-type plants (compare Figs 4 and 5 *phot2* red light). Round precipitates localized in the cytosol, at the chloroplast envelope, and the tonoplast were frequently found in the *phot2* mutant, regardless of the experimental conditions. In the double phototropin mutant, calcium precipitates after blue light were comparable with those in darkness. After red light treatment, they were much smaller and resembled those observed in wild-type plants.

### Calcium content and the area of precipitates in Arabidopsis mesophyll cells

**Fig. 6.**
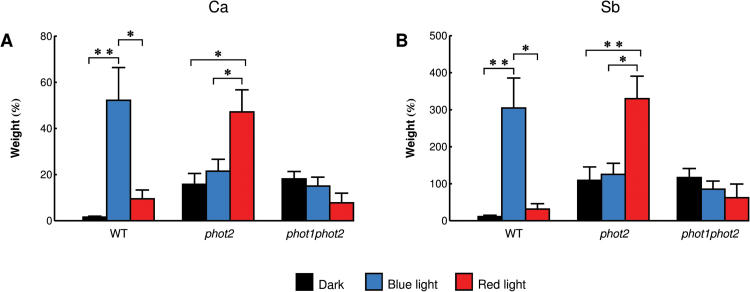
The relative content of (A) calcium and (B) antimony in precipitates at the periphery of mesophyll cells (including the cell wall and a fragment of cytoplasm) of the Arabidopsis wild type and *phot2*, *phot1phot2* mutants. The relative content of a given element in precipitates from dark-adapted leaves (black bars), leaves after blue light irradiation (blue bars), and leaves after red light irradiation (red bars). Each bar shows an average of 5–9 spectra of cell regions obtained from leaves harvested from 2–4 independent plant batches. Error bars indicate the SE. *P=0.01–0.05; **P=0.001–0.01; ***P <0.001.

To quantify the effects of light observed in the phototropin mutants, the calcium content and the area of precipitates were measured. The calcium content was determined semi-quantitatively, based on X-ray microanalysis spectra. The spectra from rectangular areas of the same size covered the periphery of a mesophyll cell (including the cell wall and a fragment of cytoplasm) lying along the cell wall (Fig. 1A). For each spectrum on the cell periphery, two control spectra were measured in regions of the vacuole and intercellular spaces. Usually small amounts of calcium were found in these control areas (results not shown). The lowest calcium content at the cell wall was in wild-type plants in darkness (Fig. 6A). Following blue light treatment, the calcium content was considerably higher than in dark conditions. This effect was also observed after red light, but was less prominent. In darkness the *phot2* and *phot1phot2* mutants had a higher calcium content in the analyzed regions than wild-type plants. In the *phot2* mutant, the amount of calcium increased only slightly after blue light treatment as compared with darkness, but it increased strongly after red light irradiation. The calcium content in the double phototropin mutant did not change significantly after blue light, as compared with darkness. Red light treatment decreased the content of calcium in this mutant, but this effect was not statistically significant. Changes in the antimony content in precipitates at the cell wall reflected those observed for calcium in all experimental groups (Fig. 6B).

**Fig. 7.**
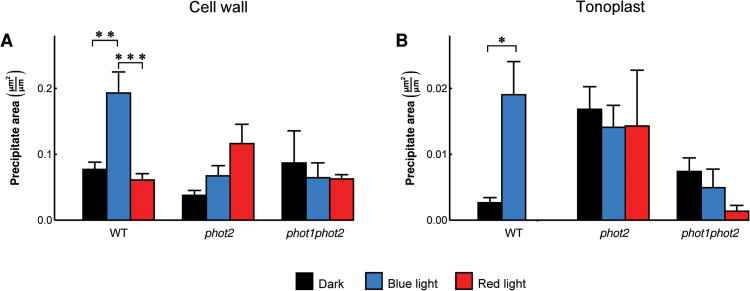
The surface area of precipitate cross-sections outside the cell walls (A) facing the intercellular space or (B) localized in the vacuole at the tonoplast in wild-type, *phot2*, and *phot1phot2* mutant Arabidopsis mesophyll cells. The area was measured in dark-adapted leaves (black bars), blue light-irradiated leaves (blue bars), and red light-irradiated leaves (red bars). Each bar shows an average of at least five images of mesophyll obtained from leaves harvested from 2–4 independent plant batches. In (B), the bar for the wild-type red light group is missing as no precipitates at the tonoplast were observed in these conditions. Error bars indicate the SE. *P=0.01–0.05; **P=0.001–0.01; ***P <0.001.

To quantify the precipitates in cell wall regions facing the intercellular space, their surface area was calculated and normalized to the cell wall length (Fig. 7A). Only the precipitates seen in contact with the cell wall were taken into account. In wild-type plants the precipitate area was larger after blue light treatment as compared with dark-adapted and red light-treated plants. The *phot2* mutant exhibited larger precipitates after red light treatment, while the difference between darkness and blue light was insignificant. In the phototropin double mutant, light conditions did not influence the area of precipitates. In all light conditions and plant lines, the precipitates in the vacuole were located mainly at the tonoplast. Their surface area was expressed per unit of tonoplast length. In wild-type plants, the surface area was significantly higher in blue light-irradiated than in dark-adapted leaves (Fig. 7B). No precipitates were observed in red light-irradiated samples. In *phot2* and the double mutant, the precipitate area was not significantly affected by light conditions.

## Discussion

Calcium imaging (for a review, see Batistic and Kudla, 2012) in mesophyll cells after light treatment presents a number of difficulties. A major obstacle to the use of fluorescence microscopy lies in the autofluorescence of cell components (Miyawaki *et al.*, 1999). The introduction of fluorescent dyes is inefficient and results in toxic effects which hamper chloroplast movements, as shown in *Lemna* (Tlałka and Fricker, 1999). The cameleon system, which relies on the sensitivity of calmodulin to calcium and on the resonance energy transfer between different green fluorescent protein (GFP) variants, is difficult to handle because the excitation wavelength overlaps with the action spectra of phototropins (Miyawaki *et al.*, 1999). The use of the aequorin system is also limited, as the detection method allows the measurement of calcium changes only after light pulses (Baum *et al.*, 1999; Harada *et al.*, 2003) and provides no direct image of calcium distribution in the cell. The KPA precipitation method chosen in this work identifies a Ca^2+^ fraction which is relatively loosely bound to cell components and visualizes the dynamics of these mobile ions after a chosen stimulus (Wick and Helper, 1982). The major disadvantage of KPA precipitation is that it does not allow changes in calcium concentration to be continuously monitored. The time resolution of the method is limited by the speed of KPA and fixative penetration in the tissue; thus, real-time investigation of calcium signaling during the onset of strong blue light is not possible. However, the combination of X- ray analysis with TEM yields more detailed information about calcium localization thanks to its higher resolution as compared with methods based on optical microscopy. In this work, the presence of calcium precipitates within the cell walls, for example in the middle lamella and at the border of the intercellular space or protoplast, was shown very precisely. The morphology of precipitates was also determined and it was confirmed that they indeed contain Ca^2+^. This would be virtually impossible using fluorescence techniques.

### Blue light-dependent calcium localization in Arabidopsis mesophyll cells

In wild-type Arabidopsis mesophyll cells in darkness, concentric calcium precipitates localized predominantly at the cell wall. Some were also found in the cytoplasm. Their positioning suggests that the flow of calcium ions takes place in both directions, both into and out of the cytoplasm. After 3min of blue light irradiation (100 μmol m^−2^ s^−1^), spherical structures at the cell wall were bigger, multilayered, and aligned in clusters. Precipitates in the cytoplasm and adjacent to the chloroplast envelope or the tonoplast were also observed. These results demonstrate that blue light causes specific calcium mobilization within the Arabidopsis mesophyll cells. However, the nature of changes in loosely bound calcium content remains obscure. The activity of membrane channels in mesophyll protoplasts after 15min of blue light (Stoelzle *et al.*, 2003) and changes in [Ca^2+^]_cyt_ after a 10s blue light pulse (Harada *et al.*, 2003) were examined in Arabidopsis leaves. In continuous blue light of a considerably lower intensity (25 μmol m^−2^ s^−1^), Ca^2+^ fluxes were analyzed using an ion-selective microelectrode only in decapitated Arabidopsis hypocotyls. In these conditions, the maximum calcium influx into the cell occurred 3min after irradiation, then calcium efflux began (Babourina *et al.*, 2002). The increase in the number of precipitates at the cell wall and the tonoplast may reflect Ca^2+^ efflux from the cytoplasm. The alignment of spherical structures which started to connect to each other after blue light irradiation supports this assumption. This efflux may be a part of calcium signal dissipation, a mechanism through which the cell sensitivity to light is sustained during constant illumination. This hypothesis is also consistent with previous results obtained on *Lemna* cells stained with Fluo-3 as a calcium indicator. Continuous, strong blue light (75.2 μmol m^−2^ s^−1^) induced an increase in Fluo-3 fluorescence in the second minute, followed by a decrease in intensity, reaching its initial value after 10min (Tlałka and Fricker, 1999).

The circular precipitates and granules observed in the cytoplasm imply the presence of vesicles rich in calcium. As blue light induces their formation, it may be speculated that a fraction of calcium undergoes exo/endocytosis during phototropin signaling. Calcium in multivesicular compartments plays a signaling role during the response to pathogens (An *et al.*, 2006). Ca^2+^-containing vesicles co-localized in the proximity of the *Mougeotia* chloroplast. This calcium store was suggested to be important for efficient chloroplast rotation (Wagner and Rossbacher, 1980; Rossbacher and Wagner, 1984). Interestingly, phot2 which is bound to the plasma membrane moves from the cytoplasm into the Golgi complex and post-Golgi structures after blue light treatment (Aggarwal *et al.*, 2014). The physiological role of this receptor trafficking remains unknown, but it might imply that phototropin is involved in formation of the calcium signature in the cytoplasm.

After equimolar red light irradiation, calcium precipitates at the cell wall were smaller and lacked the multilayered structure, but their calcium content measured near the cell wall was elevated as compared with darkness. Our results are in line with the findings of Harada *et al.* (2003) regarding a red light-induced transient increase in [Ca^2+^]_cyt_ in wild-type Arabidopsis leaves.

### The disturbance of calcium homeostasis in *phot2* and *phot1phot2* Arabidopsis mutants

In both phototropin mutants, the effect of blue light on calcium content present in wild-type plants, was abolished. Calcium precipitates lacked multilayer structures. In the *phot1phot2* mutant, precipitates rarely occurred adjacent to the chloroplast envelope and the tonoplast, where they were common in wild-type cells (Fig. 7B). Semi-quantitative analysis of the calcium content in the precipitates at the periphery of mesophyll cells confirms microscopic observations. The amount of calcium in the examined regions in both mutants was significantly higher in darkness (Fig. 6). Also the number and area of precipitates in the vacuole in darkness were higher in mutants than in wild-type plants (Fig. 7B). Both lines of evidence point to disturbed calcium homeostasis in the studied phototropin mutants and suggest that phot2 may also regulate calcium content in darkness. Phot2 can indeed actively function in cells even in the absence of light, as shown for the dark positioning of chloroplasts (Suetsugu *et al.*, 2005).

In wild-type Arabidopsis mesophyll cells, blue light causes accumulation of calcium in the examined regions. This effect is negligible in the *phot2* mutant and absent in the *phot1phot2* mutant, indicating that in strong blue light calcium transport between the symplast and apoplast depends mainly on phot2. According to Harada *et al.* (2003), phot1 controls changes in [Ca^2+^]_cyt_ in leaves only in the range of 1–50 μmol m^−2^ s^−1^ of blue light. This study confirms that blue light of 100 μmol m^−2^ s^−1^ does not affect phot1-mediated calcium signatures in Arabidopsis mesophyll, as no calcium elevation in precipitates at the cell periphery was observed in the *phot2* mutant.

Changes in calcium patterns generated after red light were observed in wild-type and *phot2* plants, but not in the double mutant. The effect of red light on loosely bound calcium has been previously reported by Tretyn *et al.* (1992), who observed an increase in the number of KPA precipitates outside the plasma membrane and in the ER cisternae after 5min red light irradiation in oat coleoptiles. This suggests that red light irradiation leads to Ca^2+^ removal from the cytoplasm. In the present study, a similar effect was observed in Arabidopsis, but its magnitude was substantial only in the *phot2* mutant. The mechanism by which the presence of phot2 reduces the red light effect on Ca^2+^ localization remains elusive. Direct interaction between phytochromes and phototropins may be involved, as Arabidopsis phyA and phot1 were shown to interact at the plasma membrane (Jaedicke *et al.*, 2012). Its physiological relevance seems likely when considering the effect of red light on the cytoskeletal organization observed in the *phot2* background. In wild-type Arabidopsis the organization of the cortical actin cytoskeleton is similar in the blue and red light-irradiated mesophyll cells (Krzeszowiec *et al.*, 2007). In contrast, in the *phot2* mutant, strong red light causes a distinct shortening of actin filaments. Thus, phot2 together with a red light photoreceptor has been suggested to control F-actin organization. Phytochrome B might be this photoreceptor, as it has been proposed to attenuate the signaling pathway leading to the chloroplast avoidance response controlled by phot2 (Luesse *et al.*, 2010). The results presented herein suggest the involvement of calcium in arranging the actin cytoskeleton under red light in the *phot2* mutant.

Calcium homeostasis is severely disturbed in the *phot1phot2* mutant, which is reflected in the lack of light-specific changes in calcium content at the periphery of mesophyll cells. This confirms the involvement of phototropins in controlling calcium levels in mesophyll cells after light treatment.

### Phototropin2-dependent chloroplast calcium patterns

Although precipitates adjacent to the chloroplast envelope and to the tonoplast were observed in several experimental groups, they were particularly abundant after blue light in Arabidopsis wild-type cells. In the double *phot1phot2* mutant, these structures were less frequently observed, thus their formation and localization seems to depend on the presence of phototropins. The lack of phot2 causes a non-specific, light-independent formation of precipitates inside the cell. This observation is consistent with the proposed role of internal calcium stores in the phot2 signal transduction pathway (Harada *et al.*, 2003). Internal Ca^2+^ stores are involved in the control of chloroplast movements, as was shown by inhibitor studies (Tlałka and Gabrys, 1993; Tlałka and Fricker, 1999; Aggarwal *et al.*, 2013*a*). It is possible that the identified calcium precipitate patterns result from a response to blue light generated by the chloroplast. The mechanism by which the chloroplast synchronizes the direction of movement with its physiological state; that is, the efficiency of photosynthesis or the photo-oxidative damage risk, is unknown. Calcium may be a good candidate because light modulates its homeostasis inside the chloroplast. A light-dependent calcium influx into the chloroplast has been reported (for a review, see Johnson *et al.*, 2006). On the other hand, a thylakoid protein, CAS (a Ca^2+^-sensing receptor) is responsible for calcium elevation in the cytoplasm (Nomura *et al.*, 2008), showing that the chloroplast may generate calcium signatures in the cell. In *Lemna*, an increase in the level of calcium in cell wall areas neighboring with chloroplasts occurs during the chloroplast avoidance response chemically induced by lead (Samardakiewicz *et al.*, 2015). In this work, almost no precipitates have been observed inside chloroplasts. This may be due to the inaccessibility of calcium to pyroantimonate precipitation in chloroplasts, where calcium is mainly bound to thylakoid membranes and stromal proteins (Stael *et al.*, 2012).

### Calcium distribution in the context of chloroplast movements

In mesophyll cells, chloroplast movements are the main phototropin-dependent responses to blue light. Several lines of evidence show that Ca^2+^ is involved in the signaling from phototropins to chloroplasts in different species (see Banaś *et al.*, 2012). This study shows that calcium relocalization after blue light treatment requires the presence of phot2, as it is absent in the *phot2* mutant. This mutant also lacks full chloroplast avoidance in strong light, since phot1 alone can trigger only residual avoidance, followed by accumulation (Luesse *et al.*, 2010; Łabuz *et al.*, 2015). Thus the observed calcium localization pattern appears to be important for eliciting the full chloroplast avoidance response.
