# Corrigendum: Blue light-dependent changes in loosely bound calcium in Arabidopsis mesophyll cells: an X-ray microanalysis study

**DOI:** 10.1093/jxb/ery007

**Published:** 2018-02-15

**Authors:** Justyna Łabuz, Sławomir Samardakiewicz, Paweł Hermanowicz, Elżbieta Wyroba, Maria Pilarska, Halina Gabryś

**Affiliations:** 1 Department of Plant Biotechnology, Faculty of Biochemistry, Biophysics and Biotechnology, Jagiellonian University, Krakow, Poland; 2 Malopolska Centre of Biotechnology, Jagiellonian University, Krakow, Poland; 3 Laboratory of Electron and Confocal Microscopy, Faculty of Biology, Adam Mickiewicz University, Poznań, Poland; 4 Laboratory of Electron Microscopy, Nencki Institute of Experimental Biology, Polish Academy of Sciences, Warsaw, Poland

*Journal of Experimental Botany*, Vol. 67, No. 13 pp. 3953–3964, 2016 doi: 10.1093/jxb/erw089

The original version of this paper contained unspecific funding information. In order to properly accredit funders, the information has been updated as follows:

In the ‘Acknowledgements’ section, the sentence “The 3D model of calcium precipitates was funded within the EU framework of FP7, Marie Curie ITN CALIPSO [GA 2013-ITN-607-607], and a grant from the Polish National Science Center [grant no. 2011/01/B/NZ3/02160].” has been changed to read “The 3D model of calcium precipitates was funded by financial resources for science in the years 2013–2017, allocated to the realization of a co-financed international project Marie Curie ITN CALIPSO [GA 2013-ITN-607-607] within the EU framework of FP7, and a grant from the Polish National Science Center [grant no. 2011/01/B/NZ3/02160].”

The authors apologise for this error.

